# Seroprevalence of hepatitis B, C, and D and associated factors in the semi-isolated Yanomami Amazonian indigenous community

**DOI:** 10.1186/s12879-023-08928-z

**Published:** 2024-01-02

**Authors:** Mariana Pinheiro Alves Vasconcelos, Juan Camilo Sánchez-Arcila, Luciana Peres, Paulo Sérgio Fonseca de Sousa, Júlio Castro-Alves, Hermano Gomes Albuquerque, Maria Cássia Mendes-Correa, Marilza Maia-Herzog, Lia Laura Lewis-Ximenez, Lívia Melo Villar, Joseli Oliveira-Ferreira

**Affiliations:** 1 Centro de Medicina Tropical de Rondônia – CEMETRON, Porto Velho, Rondônia Brazil; 2 Laboratório de Imunoparasitologia do Instituto Oswaldo Cruz – IOC/FIOCRUZ, Rio de Janeiro, Rio de Janeiro, Brazil; 3 Laboratório de Hepatites Virais do Instituto Oswaldo Cruz – IOC/FIOCRUZ, Rio de Janeiro, Brazil; 4 Instituto Nacional de Infectologia Evandro Chagas da Fundação Oswaldo Cruz – FIOCRUZ, Rio de Janeiro, Brazil; 5 Laboratório de Doenças Parasitárias do Instituto Oswaldo Cruz – IOC/FIOCRUZ, Rio de Janeiro, Brazil; 6 Departamento de Moléstias Infecciosas e Parasitárias da Faculdade de Medicina da Universidade de São Paulo – USP, São Paulo, Brazil; 7 Laboratório de Referência Nacional em Simulídeos, Oncocercose e Mansonelose, Coleção de Simulídeos do Instituto Oswaldo Cruz – IOC/FIOCRUZ, Rio de Janeiro, Brazil

**Keywords:** Viral hepatitis, HBV, HDV, HCV, Yanomami

## Abstract

**Background:**

Viral hepatitis is a significant health concern among indigenous population in the Americas. In Brazil, reports find high endemicity of HBV and HDV infections has been reported in several indigenous groups. However, few studies have documented the prevalence of HBV, HCV and HDV in the Yanomami. In this study, the prevalence of hepatitis B, C, and D serological markers and potential risk factors were investigated to provide guidance for the development of strategies aimed at reducing viral transmission in the Yanomami indigenous villages.

**Methods:**

This cross-sectional study was carried out in March 2015 and included 430 individuals from four Yanomami villages: Alapusi (*n* = 78), Castanha/Ahima (*n* = 126), Gasolina (*n* = 105), and Taibrapa (*n* = 121). A rapid test was used for detection of HBsAg and anti-HCV and chemiluminescent immunoassay for anti-HBs, anti-HBc, and anti-HDV antibodies.

**Results:**

HBsAg, anti-HBc, and anti-HBs were detected in 8.8, 45.5, and 49.4% of the participants, respectively. The estimated HBV status: current infection 9.6% (38/395); resolved infection 43.3% (171/395); vaccine immunity 20.5% (81/395), and susceptible to HBV 26.6% (105/395). Gasolina presented the lowest prevalence of HBV infection (6.5%) and the highest prevalence of vaccine immunity (26.9%). Children < 15 years old were highly susceptible to infection, as 53.1% did not have antibodies to HBV, while more than 80% of individuals over 45 years of age had been exposed to HBV. The markers for HDV were founded among 12.5% (4/32) of the HBsAg carriers. Anti-HCV was identified in all villages, with the highest prevalence in Alapusi (5.1%). Possible risk factors such as the use of piercings, tattoos, and contact with prospectors showed no statistical difference between the groups.

**Conclusions:**

Viral hepatitis B and serological markers for HCV and HDV were found to be widely distributed among the Yanomami indigenous community, while the prevalence of vaccine immunity to HBV was low. This finding reinforces the importance of promoting systematized diagnostic and vaccination strategies in indigenous communities. Our data confirm that isolated and difficult-to-reach indigenous communities lack appropriate access to diagnosis, treatment, and vaccination.

## Introduction

Viral hepatitis is an important public health problem and accounts for a significant global disease burden and high mortality. Global efforts have prioritized the elimination of viral hepatitis, which are responsible for over 1 million deaths per year [1–3].

In 2019, the World Health Organization (WHO) estimated that approximately 296 million people were chronically infected with the hepatitis B virus (HBV), 58 million with chronic hepatitis C virus (HCV), and 15 million with HBV and hepatitis D virus (HDV) [1–4].

From 2000 to 2021, Brazil confirmed 718,651 cases of viral hepatitis, corresponding to 264,640 (36.8%) cases of HBV, 279,872 (38.9%) of HCV, and 4259 (0.6%) of HDV [5]. The prevalence of HBV, HCV, and HDV is not homogenous throughout the country, and the northern region registered 73.7% of HDV cases, 14.5% of HBV, and 3.6% of HCV cases. In fact, the highest rate of HBV and HDV carriers in this region has been reported from the Brazilian Amazon [5].

In the Brazilian Amazon Basin, seroepidemiological surveys investigating viral hepatitis in Brazilian indigenous populations have reported high endemicity, morbidity, and mortality [6]. Several studies have shown that indigenous populations comprising diverse ethnic groups are among the populations with the highest prevalence of hepatitis B and D [7–14]. However, substantial regional and local variations in the prevalence of viral hepatitis exist across indigenous communities [9, 10]. Few of these studies have simultaneously assessed the prevalence of HBV and HCV, but the available data indicate that HCV infection is less common in this area [10].

The differential prevalence of HBV infection in indigenous populations may be directly related to the transmission of viral hepatitis, including environmental conditions and cultural factors such as, burrowing flea extraction with knives, scarification, bloodletting, piercing/tattooing, sexual activity at an early age, and childbirth conditions [15, 16].

According to the Brazilian Ministry of Health, the HBV detection rate in the Yanomami area was 168.1 per 100,000 inhabitants in 2015, 27.6 in 2016, and 15.6 in 2017. Indeed in 2015, the Yanomami area had the second highest HBV detection rate among indigenous populations. For hepatitis C, the detection rate was 8.2 per 100,000 inhabitants [17]. However, few studies have reported the prevalence of HBV, HCV, and HDV in the Yanomami villages [10].

In Brazil, the indigenous lands occupy an area of 106,739,926 ha and support a population of 896.9 thousand, with a significant concentration in the Amazon Forest. The land with the largest indigenous population is the Yanomami, corresponding to approximately 5% of the total number of indigenous people in Brazil [18]. The Yanomami comprise a society of semi-nomadic hunter-agriculturist people of the tropical rainforest of Northern Amazonia whose contact with non-indigenous communities has been relatively recent. Their territory is located on both sides of the border between Brazil and Venezuela, in the Orinoco-Amazon interfluvial region. In Brazil, the Yanomami population in 2023 was 29,633, distributed among 366 villages. The Yanomami territory covers 96,000 km^2^ extending from the Roraima to Amazonas states [19]. The lack or scarcity of medical care, together with an estimated 20,000 illegal miners active in their territory, have pushed the Yanomami into a desperate state [20].

In 1992, the vaccine against hepatitis B was included in the vaccination schedule for children under 5 years of age in the Brazilian Amazon region. Although universal vaccination against HBV in the indigenous population has been implemented in Brazil since 1995, cases of HBV in indigenous children born after the introduction of the vaccine are still being reported [21, 22].

The primary strategy for controlling hepatitis B and D is vaccination, while for hepatitis C it is treatment of cases, which will interrupt the chain of transmission. For such strategies to be successful, it is important to know the seroprevalence of viral hepatitis in the region. Therefore, this study aimed to estimate the prevalence of hepatitis B, C, and D and the associated risk factors, which can help in developing strategies aimed at reducing virus transmission in the Yanomami villages.

## Methods

### Study population

A cross-sectional study was conducted in four remote indigenous villages, Castanha/Ahima, Gasolina, Taibrapa, and Alapusi, in the Marari community in March 2015. Marari is one of the 37 basic health posts in the Brazilian Yanomami territory. Located in a remote region of the Amazon rainforest, the Marari health post and nearby villages are surrounded by high mountains in the state of Amazonas, bordering Venezuela. Due to the lack of roads or highways, the seasonal nature of navigation and the high cost of air transport, access to these areas is limited to small aircraft (with a capacity of three to four passengers) or boats. The villages of Castanha/Ahima and Taibrapa are close to the health post, while Gasolina and Alapusi require an additional 3–4 hours’ walk.

Two physicians among the authors performed the physical examinations to identify the clinical signs of acute infection or advanced liver disease (cirrhosis), such as jaundice, ascites, and collateral circulation. Through a pre-established questionnaire, demographic data were obtained, including age, sex, name of the village, and risk factors. Age was stratified into two groups: < 25 and > 25 years, based on the implementation of vaccination against HBV in the region, thus separating individuals born before and after the inclusion of the HBV vaccine in the vaccination schedule. The study investigated the following risk factors: piercings, tattoos, and contact with miners (challenging to establish what kind of contact). Regarding the HBV vaccine, the participants were unable to recall which vaccines they had received, including hepatitis B and additional data were inaccessible.

### Blood samples

A blood sample of 5 mL was obtained from each participant by venipuncture using vacuum blood system in 5 mL tubes containing EDTA anticoagulant (BD Vacutainer®) and processed in the field to obtain plasma. Plasma samples were stored in cryovials in a container with liquid nitrogen-LN2 and shipped to the immunoparasitology laboratory at Fiocruz where they were stored at -20 °C until thawed for serological testing in 2022.

### Serological markers

Rapid diagnostic tests (RDT) were used in the field for HBV and HCV initial screening, and detected the hepatitis B surface antigen (HBsAg, Wama) and antibodies against HCV (anti-HCV, Vikia®/Biomerieux). The rapid diagnostic tests were transported and stored at the Marari Health Centre at room temperature according to the manufacturer’s recommendations (with a range of 2 to 30 °C). The tests were performed in the field on whole blood collected on the same day in EDTA tubes according to the manufacturer’s instructions.

Stored plasma samples were further tested at Fiocruz for the hepatitis B core antibody (anti-HBc), (LIAISON® Anti-HBc – Diasorin), hepatitis B surface antibody (anti-HBs) (LIAISON® XL Anti-HBs II - Diasorin), and anti-delta virus antibody (anti-HDV) (LIAISON® XL Anti-HDV – DiaSorin) using a chemiluminescent immunoassay (CLIA) according to the manufacturer’s guidelines. The anti-HBs results were reported in titers (mIU/mL), and protective titers were considered greater than 10 mIU/mL. Samples that were either 10% above or 10% below the established cut-off value were considered to be indeterminate result. Sample was borderline and/or indeterminate results were retested.

### Serological analysis

To study the hepatitis B marker status, four groups were identified: HBV infection, defined as the presence of the HBsAg (HBV infected individuals); resolved infection, defined as the presence of anti-HBc in the absence of HBsAg (individuals with spontaneous cure after prior contact with HBV); immune due to vaccination, defined as the presence of anti-HBs in the absence of HBsAg and anti-HBc (individuals with immunity due to HBV vaccine), and susceptible, defined as the absence of all tested HBV markers (negative for HBsAg, anti-HBc, and anti-HBs) [23].

The hepatitis B virus (HBV) status was determined in 395 out of 430 samples. The 35 specimens were excluded (five in Alapusi, seven in Castanha/Ahima, 12 in Gasolina and 11 in Taibrapa) due to indeterminate result (*n* = 5) and insufficient specimen volume to test for various serological markers used to identify HBV status (*n* = 30),

### Statistical analysis

Data were compiled in Excel spreadsheets, and statistical analysis and graph generation were performed using SPSS software (IBM-SPSS Inc., Chicago, IL, USA), the statistical software R version 4.0.2, and Graph Pad PRISM® version 8.0. For a demographic description of individuals from the Yanomami villages, we used medians and interquartile intervals for continuous numerical variables and absolute numbers and relative frequencies for nominal variables. To determine factors associated with the risk of HBV or HDV infection, resolved infection (inactive), or vaccine immunity, we used the non-parametric Mann–Whitney test to compare the continuous numerical variables and Pearson’s chi-square test or Fisher’s exact test, when necessary, to compare the nominal qualitative variables.

We used multivariate logistic models with risk of HBV infection, resolved infection (inactive), or vaccine immunity as the outcomes, and the models were adjusted for age, local (villages), and risk factors. Measures of association were presented as adjusted odds-ratios (aOR) and their 95% confidence intervals (CI). *p* < 0.05 was considered statistically significant. The map with georeferencing was produced using the free software QGIS 3.28.

## Results

### Demographic characteristics of the study population

A total of 430 individuals were included in the study from four villages of the Yanomami community in Marari: 18.1% (78/430) from Alapusi, 29.3% (126/430) from Castanha/Ahima, 24.5% (105/430) from Gasolina, and 28.1% (121/430) from Taibrapa (Table 1). The median age of the participants was 26 ± 17.2 years. The Yanomami villages had a relatively uniform gender (*p* = 0.22) and age (*p* = 0.56) distribution. Regarding risk factors for viral hepatitis, 95.1% had piercings, 63.9% had tattoos, and 33.9% had contact with miners.

**Table 1:** Absolute and relative frequencies of demographic variables and risk factors among individuals from Yanomami, Brazil

		Yanomami villages					
		Alapusi	Castanha/Ahima	Gasolina	Taibrapa	Total	*p*
		*n* = 78	*n* = 126	*n* = 105	*n* = 121	*n* = 430	
		n (%)	n (%)	n (%)	n (%)	n (%)	
**Gender**	Male	31 (39.7)	62 (49.2)	39 (37.1)	48 (39.7)	180 (41.9)	0.22
	Female	47 (60.3)	64 (50.8)	66 (62.9)	73 (60.3)	250 (58.1)	
**Age group**^a^ (years)	0–15	19 (24.4)	17 (13.5)	24 (22.9)	34 (28.1)	94 (21.9)	
	16–30	27 (34.6)	51 (40.5)	36 (34.3)	36 (29.8)	150 (34.9)	
	31–45	15 (19.2)	26 (20.6)	21 (20.0)	28 (23.1)	90 (20.9)	0.56
	46–60	10 (12.8)	20 (15.9)	16 (15.2)	13 (10.7)	59 (13.7)	
	> 60	6 (7.7)	8 (6.3)	8 (7.6)	10 (8.3)	32 (7.4)	
**Year of Birth**	Before 1995	33 (42.3)	52 (41.3)	53 (50.5)	60 (49.6)	198 (46.0)	
	After 1995	44 (56.4)	70 (55.5)	52 (49.5)	61 (50.4)	227 (52.8)	0.76
**Risk Factors**	Piercing	72 (92.3)	121 (96.0)	100 (95.2)	116 (95.9)	409 (95.1)	0.34
	Tattoo	43 (55.1)	88 (69.8)	75 (71.4)	69 (57.0)	275 (63.9)	0.01
	Contact/ miners	29 (37.2)	43 (34.1)	49 (46.7)	25 (20.7)	146 (33.9)	< 0.01

^a^Age not provided for 10 samples; before 1995: individuals born before HBV vaccine implementation and after 1995: individuals born after HBV vaccine implementation. *p* < 0.05 was considered statistically significant

### Prevalence of viral hepatitis serological markers

All 430 individuals were tested for HBsAg, and 376 were additionally tested for anti-HBc, 285 for anti-HBs, and 87 for anti-HDV. The overall prevalence of HBsAg was 8.8% (38/430), ranging from 11.6% in Taibrapa to 5.7% in Gasolina. As for anti-HBs, isolated or associated with anti-HBc, we found a prevalence of 49.4% (141/285), ranging from 57.3% in Castanha/Ahima to 43.6% in Gasolina; for anti-HBc, we found a prevalence of 45.5% (171/376), ranging from 55.7% in Castanha/Ahima to 32.6% in Gasolina. Anti-HDV testing was performed in 32 out of 38 samples from individuals with HBV infection and in 55 out of 171 samples that were anti-HBc positive/HBsAg negative (resolved infection). The positivity rate for anti-HDV was 12.5% (4/32) among the HBsAg carriers and 7.3% (4/55) among individuals with resolved infection. The four participants that tested positive for HBV/HDV were women, and two out of the six HBsAg positive samples (33.3%) from Gasolina were coinfected with HDV. None of the individuals had clinical signs of acute infection or advanced liver disease (cirrhosis). Despite the high numbers of anti-HDV positive individuals, the sample size was too small to carry out a statistical comparison between the villages (Table 2).

**Table 2:** Absolute and relative frequencies of serological markers for viral hepatitis among individuals from Yanomami, Brazil

Serological marker	Yanomami villages				
	Alapusi	Castanha/Ahima	Gasolina	Taibrapa	Total
	Pos/n (%)	Pos/n (%)	Pos/n (%)	Pos/n (%)	Pos/n (%)
**HBsAg**	7/78 (8.9)	11/126 (8.7)	6/105 (5.7)	14/121 (11.6)	38/430 (8.8)
**Anti-HBs**	25/49^b1^ (51.0)	43/75^b1^ (57.3)	31/71^b1^ (43.6)	42/90^b2^ (46.7)	141/285 (49.4)
**Anti-HBc**	34/69 (49.3)	63/113 (55.7)	29/89 (32.6)	45/105 (42.8)	171/376 (45.5)
**Anti-HCV**	4/78 (5.1)	2/126 (1.6)	1/105 (0.9)	2 /121 (1.6)	9/430 (2.1)
**Anti-HDV**^a^					
**HBsAg+**	0/6 (0)	0/10 (0)	2/6 (33.3)	2/10 (20.0)	4/32 (12.5)
**Anti-HBc+**	1/2 (50.0)	0/19 (0)	1/7 (14.3)	2/27 (7.4)	4/55 (7.3)

*Pos/n* Number of positives/number of sample tested, ^a^*HBSAg+* anti-HDV and HBsAg positive, *anti-HBc+* Anti-HDV and Anti-HBc positive and HBsAg negative, ^b^number of indeterminate result

Anti-HCV was identified in all villages with the highest prevalence found in Alapusi (5.1%), and among the positives (9/78), four were women and five men. The median age was 38 ± 19.2 years, compared to 26 ± 17.1 years for anti-HCV negative individuals. No familial link was found among the anti-HCV positive cases (Table 2).

### Hepatitis B serological marker status

**Fig. 1:**
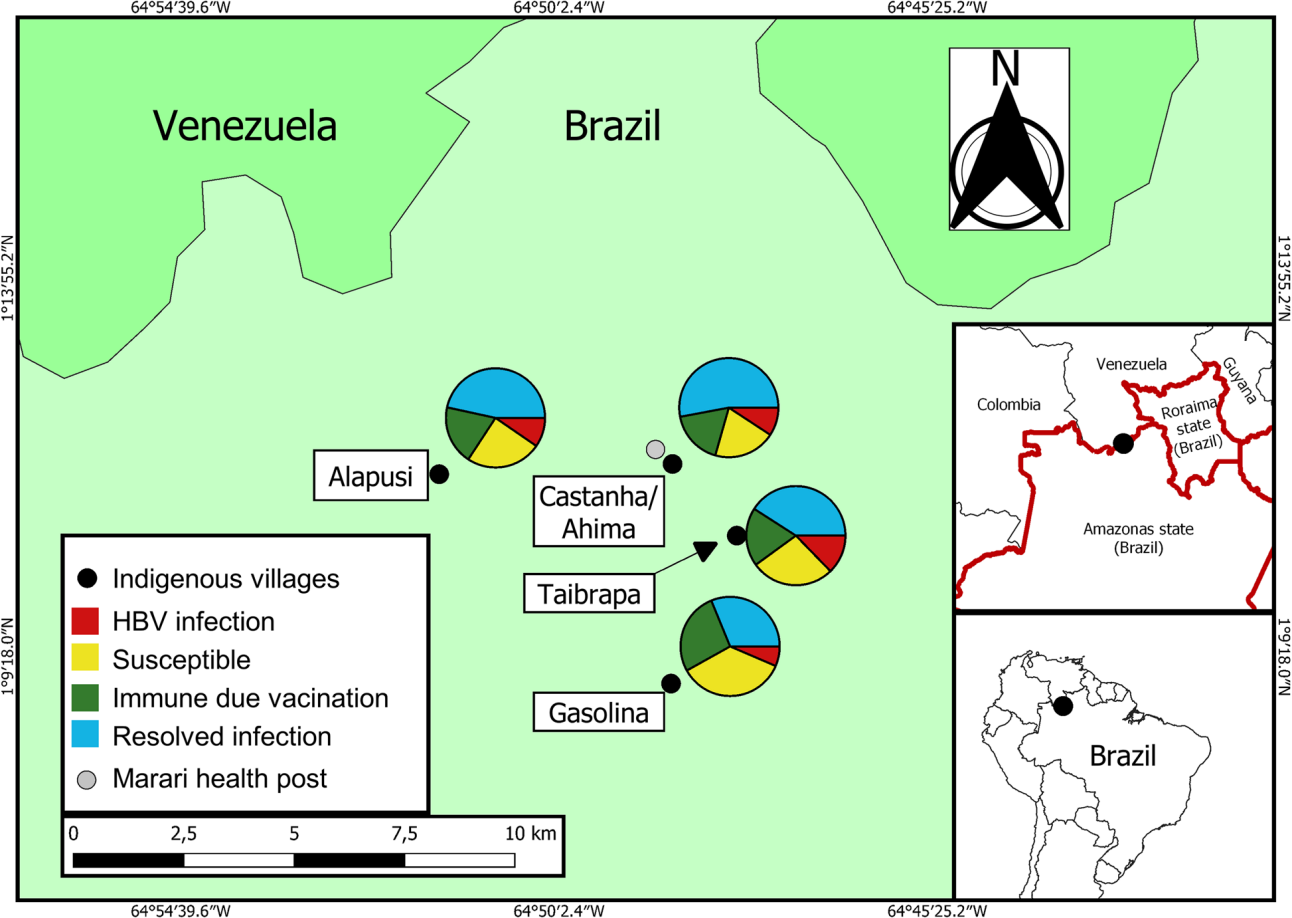
Map of the Brazilian-Venezuelan border showing the study area and the HBV marker status

Regarding the hepatitis B markers, Fig. 1 shows the location of the Marari Health post, the Yanomami villages, and HBV status according to the villages. To analyzed HBV status, we evaluate 395 individuals. HBV infection was higher in Taibrapa (12.7%), while resolved past infection was higher in Castanha/Ahima (52.9%). Gasolina presented the lowest prevalence of HBV infection (6.5%) and the highest prevalence of vaccine immunity (26.9%). However, the prevalence of susceptible individuals ranged from 35.5% in Gasolina to 20% in Castanha/Ahima.

Table 3 shows the seropositivity for HBV according to specific groups. The prevalence of HBV infection, resolved infection, vaccine immunity, and susceptibility in the population was 9.6% (38/395), 43.3% (171/395), 20.5% (81/395), and 26.6% (105/395), respectively. Men and women did not differ markedly in the rates of positivity for HBV serological markers (*p* = 0.23).

**Table 3:** HBV marker status, demographics, and risk factors among individuals from Yanomami, Brazil

	HBV Infection	Resolved Infection	Vaccine immunity	Susceptibility	*p*
	n(%)	n(%)	n(%)	n(%)	
**Overall**	38 (9.6)	171 (43.3)	81 (20.5)	105 (26.6)	
**Gender**					
Female	22 (9.4)	94 (40.2)	55 (23.5)	63 (26.9)	0.23
Male	16 (10.0)	77 (48.1)	25 (15.6)	42 (26.3)	
**Age group (years)**					
0–15	9 (11.1)	2 (2.5)	27 (33.3)	43 (53.1)	< 0.01
16–30	14 (10.0)	38 (27.2)	36 (25.7)	52 (37.1)	
31–45	7 (8.0)	68 (78.2)	9 (10.3)	3 (3.5)	
+45	8 (9.6)	62 (74.7)	7 (8.4)	6 (7.2)	
**Year of Birth**					
After 1995	19 (10.4)	17 (9.3)	58 (31.7)	89 (48.6)	< 0.01
Before 1995	19 (9.1)	153 (73.6)	21 (10.1)	15 (7.2)	
**Risk Factor**					
Piercing	37 (9.9)	169 (45.1)	74 (19.7)	95 (25.3)	0.05
Tattoo	23 (8.8)	116 (44.6)	55 (21.2)	66 (25.4)	0.52
Contact/miners	18 (13.2)	61 (44.9)	21 (15.4)	36 (26.5)	0.18

After 1995: individuals born after HBV vaccine implementation and before 1995: individuals born before HBV vaccine implementation. *p* < 0.05 was considered statically significant

Regarding the age distribution of HBV markers, HBV infection was detected in all age groups. Children < 15 years old were highly susceptible to infection, as 53.1% did not have antibodies to HBV, while more than 80% of individuals over 45 years of age had been exposed to HBV. The mean age among HBV infected individuals was 25 years (IQR 17;39), while it was 39 years (IQR 31;53) in those with resolved infection, 21 years (IQR 14;27) in those with vaccine immunity, and 17 years (IQR 13;22) in the susceptible group. Being born before or after the implementation of vaccination was also statistically significant when comparing the HBV groups. Among those born before HBV vaccine implementation, there was a much higher prevalence of resolved infection, while those born after had a higher proportion of susceptible individuals (Table 3). Possible risk factors such as the use of piercings, tattoos, and contact with prospectors showed no statistical difference between the groups.

### Exposed and unexposed to HBV

Individuals in the age group of 16–30 years were 3.9 times more likely to have been exposed to HBV than the group aged 0–15 years (*p* < 0.01). Individuals in the 31–45 years age-group had the highest chance of being exposed to HBV. Compared to the Castanha/Ahima villages, which are close to the health post, individuals from the Gasolina village were 78% less likely to be exposed to HBV. Gender, piercing, tattooing, and contact with miners were not associated with exposure (Table 4).

**Table 4:** Results of the adjusted logistic models for exposure to HBV in individuals from Yanomami, Brazil

	Exposed to HBV			
	No (*n* = 186)	Yes (*n* = 209)	aOR [95%CI]	*p*
**Gender**				
Female	118 (50.4)	116 (49.6)	Ref.	
Male	67 (41.9)	93 (58.1)	1.05 [0.42;2.66]	0.91
**Villages**				
Castanha/Ahima	45 (37.8)	74 (62.2)	Ref.	
Alapusi	32 (43.8)	41 (56.2)	0.85 [0.36;1.98]	0.70
Gasolina	58 (62.4)	35 (37.6)	0.22 [0.1;0.49]	< 0.01
Taibrapa	51 (46.4)	59 (53.6)	0.74 [0.36;1.53]	0.41
**Age group (years)**				
0–15	70 (86.4)	11 (13.6)	Ref.	
16–30	88 (62.9)	52 (37.1)	3.91 [1.65;10.01]	< 0.01
31–45	12 (13.8)	75 (86.2)	43.19 [15.73;132.76]	< 0.01
+ 45	13 (15.7)	70 (84.3)	28.04 [10.41;83.60]	< 0.01
**Risk factors**				
Piercing				
No	7 (87.5)	1 (12.5)	Ref.	
Yes	169 (45.1)	206 (54.9)	2.06 [0.27;44.2]	0.54
Tattoo				
No	59 (51.8)	55 (48.2)	Ref.	
Yes	121 (46.5)	139 (53.5)	0.87 [0.4;1.81]	0.71
Contact/miners				
No	121 (48.8)	127 (51.2)	Ref.	
Yes	57 (41.9)	79 (58.1)	1.54 [0.66;3.61]	0.32

*Exposed to HBV* current (HBsAg positive) and/or past infection (HBsAg negative/anti-HBc positive); Logistic model adjusted for gender, age group, and risk factors; *aOR* adjusted odds ratio, *CI* confidence interval

## Discussion

This study was carried out 24 years after the implementation of vaccination against HBV in the Yanomami community; despite this initiative, this population continues to have high HBV endemicity [23, 24]. We found a prevalence rate of 50.4% (208/413) for HBV exposure (present or past infection) in this indigenous population. HBV infection was higher in Taibrapa (12.7%), while resolved infection was higher in Castanha/Ahima (52.9%; *p* < 0.01). Gasolina presented the lowest prevalence of HBV infection (6.5%) and the highest prevalence of immunity due to vaccination (26.9%). The HBV detection rate in the Yanomami population reported by the Ministry of Health was also high in 2015 (168.1 per 100,000 inhabitants), with a drastic decrease in 2016 (27.6) and 15.6 in 2017 [17]. However, the data reported do not specify which Yanomami villages were surveyed, and the lower detection rate in 2016 and 2017 could be due to different villages being surveyed. Previous studies, including ours, have shown that the prevalence of HBsAg carriers is heterogeneous, even between villages located in very close proximity and with very similar cultural, social, and economic identities.

Earlier studies carried out in indigenous populations before the implementation of HBV vaccination reported extremely high prevalence rates of HBV infection (7.3–30.6%) [15, 25]. Studies after the implementation of HBV vaccination in the indigenous population showed that the prevalence was also high in the Yanomami communities of Venezuela, with rates of 14.3% for HBsAg and 58% for anti-HBc [16]. In the Brazilian Amazon, moderate endemicity was found; the prevalence of previous HBV infection was 55.7%, with 5.4% chronic carriers (HBsAg) in the indigenous of Apyterewa, while the prevalence was 49.5% with 1.1% carriers in the indigenous of Xingu villages. This pattern was also detected among ethnic groups of the Amazonas State [6]. In Warao Amerindians from Venezuela, the prevalence of HBsAg was 1.8% and that of anti-HBc was 13% [26].

In non-indigenous areas of the Amazonas state, the prevalence of HBsAg prior to the introduction of the HBV vaccine was 16.7% [27]. However, 11 years after vaccine introduction, the prevalence of HBsAg in the region was 3.3% and that of anti-HBc was 49.9% [22]. Unfortunately, the Yanomami did not display these improvements following the implementation of HBV vaccination to prevent and control this infection.

Previous studies in highly endemic areas have demonstrated that cultural practices may be associated with the chain of transmission of viral hepatitis, for example, scarification, bloodletting, piercing, tattooing, sexual activity at an early age, oral exposure via breastfeeding, sharing prechewed food and tobacco, and consumption of drinks fermented with saliva [11, 16].

We found that the use of earrings or nose rings, scarifications, and tattoos are common practices in the Yanomami community and tattooing could be one of the factors associated with previous HBV infections. Yanomami women usually wear ornaments in three holes around the lips and in the nasal septum, with rods cut from stems or roots, while the men often have holes in their earlobes [19]. Tattoos including scarifications are also frequently observed in this population.

In the present study, 26.6% of the individuals were susceptible to HBV and less than 21% had immunity against HBV, indicating that few individuals are protected against HBV infection, but according to the Indigenous Health Secretariat (SESAI), vaccination coverage in the indigenous population in 2011, 2014, and 2018 for HBV was greater than 90% [17]. However, the prevalence of susceptible individuals requiring vaccination in our study ranged from 35.5% in Gasolina to 20% in Castanha/Ahima. Another interesting finding is the fact that most susceptible individuals were identified among those born after the vaccination was implemented (≤25 years). Previous studies have shown that after immunization, the concentration of anti-HBs decreases over time, with approximately 15 to 50% of vaccinated children having anti-HBs concentrations < 10 mIU/mL 5 to 15 years after vaccination [28]. In addition, approximately 30 to 60% of vaccinated adults will have anti-HBs concentrations reduced to < 10 mIU/mL in approximately 10 years [29, 30]. These data may explain the very low prevalence of vaccine immunity in the Yanomami population. Due to the cultural habits of the Yanomami people, living in a remote area, and being semi-nomadic, vaccination in these villages has several challenges.

The low prevalence of vaccine antibodies may be due to the difficulty in administering the three doses, storing and transporting vaccines in temperature controlled condition over great distances, or even the agreement of the indigenous people, including the mothers, to let their newborns be vaccinated [16]. Breaking the chain of transmission, ensuring maternal diagnosis and treatment, immunoglobulin administration, and vaccination of the newborn should be prioritized [31].

As almost 50% of this population and 80% of individuals aged ≥25 years have already been exposed to HBV, surveillance and revaccination are needed, given the susceptibility to HBV and the lack of reliable vaccination records. Since these data were collected, HBV vaccination of susceptible individuals has been performed in this area.

Worldwide, it is estimated that approximately 4.5% (95% CI 3.6–5.7) of all HBsAg-positive people are co-infected with HDV [32]. In our study, the prevalence was higher (12.5%), similar to the overall rate of HDV infection reported in seven indigenous populations in the Amazon basin (13.4%) in Brazil [9].

Chronic hepatitis D is the most severe and rapidly progressive form of all chronic viral hepatitis [33]. However, in our study no individuals coinfected with HDV demonstrated clinical signs compatible with acute infection or advanced liver disease (cirrhosis).

Hepatitis B and D do not have an effective curative treatment. The treatment, when indicated, aims to suppress the viral load and needs to be continued indefinitely. Although our study did not identify any individuals with clinical signs of advanced liver disease (cirrhosis), HBV is a known oncogenic virus and chronic HBV infection, even in individuals without cirrhosis, increases the risk of hepatocellular carcinoma (HCC) [34]. Screening for HCC should be done periodically with ultrasound [35]. In the Yanomami indigenous population, this screening becomes complicated due to difficult access, lack of energy for the device, and difficulty for a qualified professional to visit the villages periodically.

Regarding the prevalence of anti-HCV, this study found an overall prevalence of 2.1%. Previous studies have generally described low prevalence of HCV in the Amazon region. HCV was not detected in indigenous Warao from Venezuela [26] and in non-Amazonian indigenous peoples from Mato Grosso do Sul – Brazil [36]. The prevalence in Amerindians from Tocantins – Brazil was 1.2% [13]. For the treatment of hepatitis C, currently direct action antivirals (DAAs) are used, with an average duration of 12 weeks, with few adverse effects and a cure rate greater than 95% [37]. Thus, efforts must be made to eliminate HCV in indigenous villages. Unfortunately, HCV RNA testing was not performed due to issues with sample availability.

This study has some limitations. It was not possible to evaluate the viral load of individuals positive for any of the hepatitis, which makes impossible to diagnose active infection in individuals with positive anti-HCV and anti-HDV. Given the small amount of the blood sample and impossibility of new blood draws, we chose a screening strategy in this specific population that made it impossible to evaluate isolated anti-HBc, as well as to evaluate anti-HDV in all samples positive for anti-HBc. Blood collection was only authorized by the research ethics committee for participants aged over 10 years. The language barrier hindered a more detailed clinical history pertaining to past signs and symptoms of hepatitis, as well as the epidemiological factors. Laboratory tests (hematological and biochemical) and abdominal ultrasonography could have substantially contributed to a better assessment of liver disease in individuals that tested positive.

## Conclusion

Viral hepatitis B and serological markers of HCV and HDV were found to be widely distributed among the Yanomami indigenous community, while the prevalence of vaccine immunity to HBV was low. Testing for serological markers of these infections and constant surveillance strategies must be emphasized for appropriate monitoring of HBV vaccine coverage and early detection and treatment of hepatitis cases. The elimination of viral hepatitis is a formidable challenge, and our data confirm that isolated and difficult-to-reach indigenous communities lack appropriate access to diagnosis, treatment, and vaccination. These findings reinforce the importance of promoting systematized diagnostic and vaccination strategies in indigenous communities. It is worth noting that HBV vaccination not only protects against HBV but also HDV infection.

## Data Availability

The datasets generated and/or analyzed during the current study are available from the corresponding author on reasonable request.

## Abbreviations

aOD: Adjusted odds-ratios
CI: Confidence Interval
DAA: Direct action antivirals
HBV: Hepatitis B virus
HCC: Hepatocellular carcinoma
HCV: Hepatitis C virus
HDV: Hepatitis B vírus
SESAI: Indigenous Health Secretariat
WHO: World Health Organization
